# Serum lipids, oxidative stress, and systemic inflammation mediate the association between circadian syndrome and periodontitis

**DOI:** 10.3389/fnut.2025.1622348

**Published:** 2025-07-02

**Authors:** Ruoyao Zhang, Chong Han, Dijia Hu, Qiukai Chen, Jinguo Zheng, Jiangshan Chen, Toshinori Okinaga

**Affiliations:** ^1^Department of Microbiology, Graduate School of Dentistry, Osaka Dental University, Hirakata, Osaka, Japan; ^2^Second Department of Oral and Maxillofacial Surgery, Graduate School of Dentistry, Osaka Dental University, Hirakata, Osaka, Japan; ^3^Department of Operative Dentistry, Graduate School of Dentistry, Osaka Dental University, Hirakata, Osaka, Japan; ^4^Department of Microbiology, Osaka Dental University, Hirakata, Osaka, Japan

**Keywords:** circadian rhythm, periodontitis, metabolic syndrome, circadian syndrome, NHANES

## Abstract

**Background:**

Circadian rhythm disruption is an underlying cause of metabolic syndrome (MetS) and is associated with the development of periodontitis. Circadian syndrome (CircS) is an emerging measure of circadian rhythm disruption based on MetS. We aimed to explore the cross-sectional association between CircS and periodontitis through the National Health and Nutrition Examination Survey 2009–2014.

**Methods:**

We included 7,555 eligible participants. CircS was assessed by the five components of the MetS, depression, and short sleep duration, with fulfillment of ≥4 of the 7 components indicating the presence of CircS. Periodontitis was evaluated according to the Centers for Disease Control and Prevention-American Academy of Periodontology criteria. Multivariable logistic regression analysis was used to explore the association between CircS and periodontitis. In addition, restrictive cubic spline analysis, mediation analysis, and stratified analysis were used to comprehensively evaluate these associations.

**Results:**

After adjusting for all confounders, CircS was significantly associated with periodontitis (odds ratio 1.509, 95% CI 1.326–1.716, *p* < 0.0001). Similar findings were found for CircS components. A higher number of components was associated with increased odds of periodontitis compared to participants without any components. However, among CircS participants, higher components were not associated with the odds of periodontitis. The number of CircS components was nonlinearly associated with periodontitis, and a positive association existed only when the number of components was <4. Mediation analyses suggested that several serum lipids, oxidative stress, and systemic inflammation markers mediated the association of CircS with periodontitis. This association was more pronounced in participants <60 years of age, income-poverty ratio >3, and non-vigorous physical activity.

**Conclusion:**

The presence of CircS was significantly associated with increased odds of periodontitis, serum lipids, oxidative stress, and systemic inflammation may mediate this association. These findings emphasize that CircS may serve as an independent risk factor for periodontitis and provide insights for individualized prevention of periodontitis.

## 1 Introduction

Periodontitis is a chronic inflammatory disease affecting the supporting tissues of the teeth that is triggered by bacteria in the plaque biofilm, leading to progressive destruction of the periodontal ligament and alveolar bone (1). Periodontitis is one of the most common oral diseases and is associated with a significant increase in the burden of oral disease. Reports from the Global Burden of Disease Study 2021 suggest that severe periodontitis is one of the most common oral diseases (age-standardized prevalence 12,500/100,000 persons), with more than 1 billion people worldwide suffering from severe periodontitis, and is associated with one of the highest disability-adjusted life-year burdens (2, 3). A nationally representative cross-sectional survey showed that periodontitis affects 42% of adults in the United States, with 7.8% having severe periodontitis (4). In addition, many observational studies have revealed that periodontitis may act as a systemic disease and is associated with an increased risk for the development of multiple complications, such as cardiovascular disease (CVD), diabetes, and adverse pregnancy outcomes, with possible bidirectional associations (5–8). Understanding the modifiable risk factors associated with periodontitis and implementing relevant individualized preventive strategies is essential to reduce the burden of periodontitis.

Metabolic syndrome (MetS) refers to a combination of cardiometabolic risk factors including dysglycemia, atherogenic dyslipidemia, hypertension, and central obesity (9). MetS is a major chronic non-communicable condition that affects approximately one quarter of the adult population worldwide (10). A large body of epidemiologic research has demonstrated a strong bidirectional association between MetS and periodontitis. A recent meta-analysis showed that moderate and severe periodontitis were significantly associated with the development of MetS (11). Another meta-analysis showed a significant bidirectional association between periodontitis and MetS overall after pooling the results, although there was a lack of correlation in women (12). In addition, a meta-analysis demonstrated a dose–response association between the number of MetS components and the development of periodontitis, i.e., as the number of components increased, the magnitude of the effect increased (13). Circadian rhythms are physiological and behavioral rhythms controlled by the biological clock in an organism which regulates a wide range of physiological functions and can be calibrated by external environmental signals (14). In addition to MetS, accumulating evidence suggests that circadian rhythm disruption may play an important role in the onset and progression of periodontitis (15). Experimental evidence suggests that many circadian rhythm gene expressions show significant reductions in periodontitis, and that circadian rhythm disruption may contribute to periodontitis progression by promoting inflammatory infiltration of immune cells and alveolar bone loss (16–18).

Notably, a large body of evidence suggests a strong association between metabolic disorders and circadian rhythms as well (19). Circadian disruption alters the gut microbiome and disrupts metabolic homeostasis and inflammatory pathways leading to MetS (20). Recently, studies have combined MetS with circadian rhythm disorders to emphasize the close association and crosstalk between them and proposed the circadian syndrome (CircS) (21). CircS combines short sleep duration and depressive symptoms that can negatively impact circadian rhythms to more fully represent the strong association between circadian misalignments and metabolic disorders (22). Recent clinical evidence suggests that CircS is associated with the risk of several chronic diseases such as CVD and cognitive decline and may have better predictive value compared to MetS (22–24). However, the association between CircS and periodontitis still lacks real-world exploration. Elucidating this association could help to reveal CircS as a modifiable and practically relevant risk factor for periodontitis prevention and management in the general population and to identify high-risk groups for periodontitis associated with circadian rhythm disorders, which could then be targeted for focused attention and early intervention.

Here, we used nationally representative data from the National Health and Nutrition Examination Survey (NHANES) to examine the association of CircS and its components with periodontitis in the general population. It is worth noting that the NHANES 2009–2014 data used in this study has unique advantages, including the first-ever full-mouth dental assessment in periodontal examinations and the simultaneous collection of sleep and depression symptom indicators. In terms of methodology, weight adjustments for complex sampling ensured that the sample was nationally representative. In addition, we reveal the potential role of lipid profiles, oxidative stress, and systemic inflammation in this association. We used multivariable logistic regression models to analyze the association between CircS and periodontitis. In addition, we used restricted cubic splines (RCS) to examine the dose–response relationship between the number of CircS components and periodontitis and employed mediation analysis to investigate the potential pathways of serum biomarkers.

## 2 Methods

### 2.1 Study design and population

NHANES uses a complex cross-sectional survey design to collect data from the U. S. non-institutionalized population in biennial cycles, with the goal of providing a comprehensive understanding of the health and nutritional status of the U. S. population at a given point in time. To ensure that the sample is representative, NHANES employs a stratified multi-stage probability sampling method to select survey respondents, ensuring that the sample covers a wide range of ages, sexes, races, ethnicities, and other groups. As a principal project of the National Center for Health Statistics (NCHS), all protocols from NHANES have been approved by the NCHS Ethics Review Board (Continuation of Protocol #2005–06 and (Continuation of) Protocol #2011–17) and all participants provided written informed consent.

**Figure 1:**
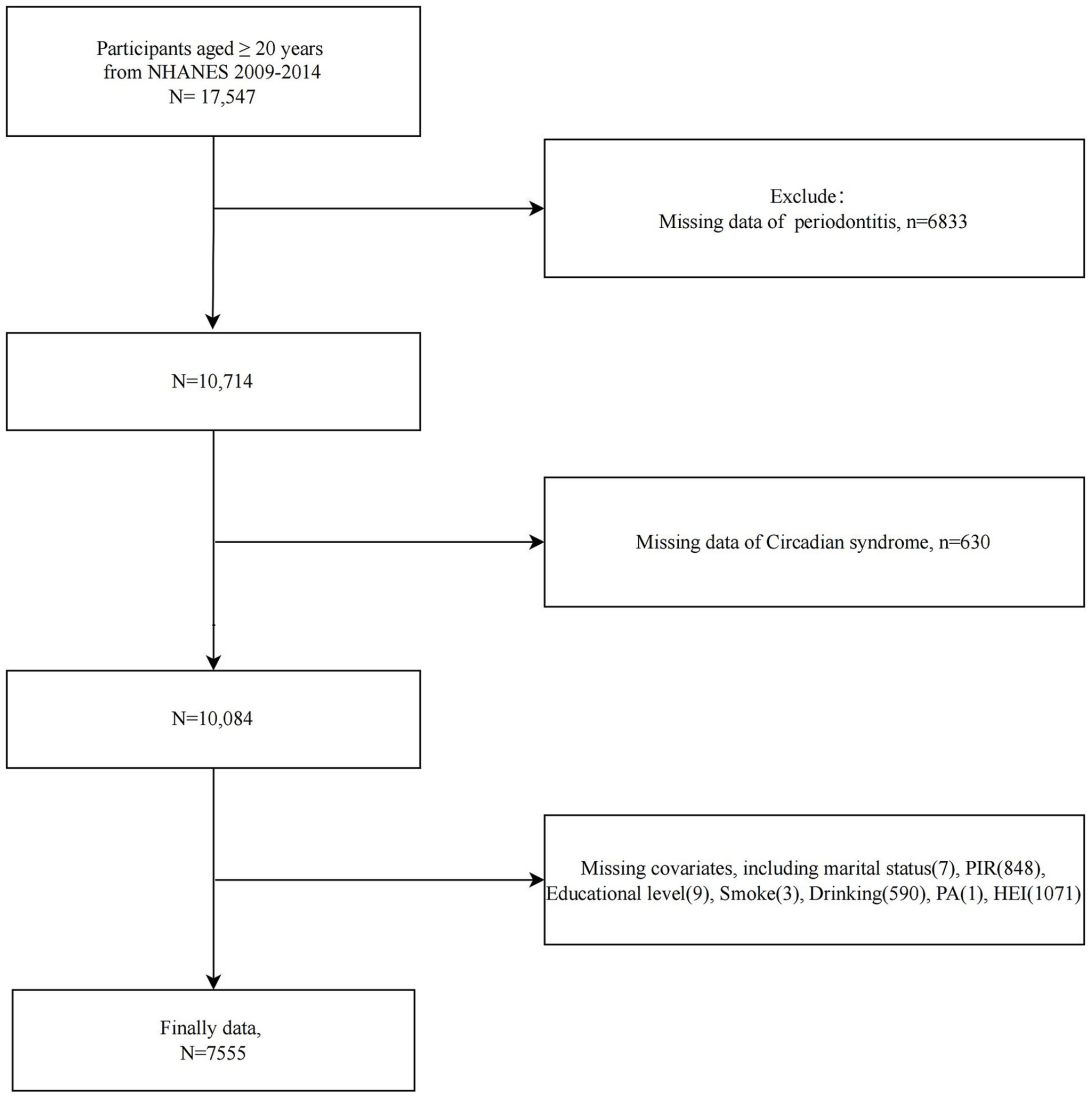
Flowchart of study population selection, NHANES 2009–2014.

The study population selection flowchart was presented in Figure 1. We first included all participants ≥20 years of age from NHANES 2009–2014 (*n* = 17,547). Participants with missing information on periodontitis (*n* = 6,833), missing CircS (*n* = 630), and missing major covariates (*n* = 2,529) were excluded. A total of 7,555 participants were included in further analyses (*n* = 7,555).

### 2.2 Assessment of CircS

According to previous studies, the presence of CircS was indicated by meeting at least 4 of 7 criteria: (1) waist circumference (WC) ≥ 102 cm/88 cm in men/women; (2) serum triglycerides (TG) ≥ 150 mg/dL or use of lipid-lowering drugs; (3) serum high-density lipoprotein-cholesterol (HDL-C) < 40/50 mg/dL in men/women or use of lipid-lowering drugs; (4) fasting blood glucose (FBG) ≥ 100 mg/dL or use of glucose-lowering drugs; (5) mean blood pressure testing ≥130/85 mmHg or use of antihypertensive drugs; (6) sleep duration ≤6 h (short sleep duration); and (7) depression symptom score ≥10 as assessed by Patient Health Questionnaire-9 (PHQ-9) (25–28). WC was determined by standard methods by trained staff at a mobile examination center (MEC). WC was measured at the midpoint between the iliac crest and the lower rib margin using a non-stretchable tape, with participants standing and breathing normally. Two measurements were averaged, following NHANES standardized protocols. Serum lipid profiles were obtained from standard biochemical files from laboratory examinations. FBG was determined by examining blood specimens collected in the morning after an 8 h fast. Sleep duration and PHQ-9 were obtained from participant self-reports in the relevant questionnaires.

### 2.3 Evaluation of periodontitis

A periodontist examined six positions of each tooth (excluding the third molar) of eligible participants (aged 30 years and older and without any health concerns) at the MEC. Participants’ periodontal examination profiles were obtained from the Oral Health-Periodontal section of NHANES and consisted of gingival recession (GR), pocket depth (PD), and attachment loss (AL, defined as the difference between PD and GR). The definition of periodontitis in this study followed the Centers for Disease Control and Prevention-American Academy of Periodontology (CDC-AAP) criteria for monitoring periodontitis (29). Mild periodontitis was categorized by having at least two interproximal sites with AL ≥ 3 mm alongside PD ≥ 4 mm on different teeth, or a single site with PD ≥ 5 mm. Moderate periodontitis was characterized by at least two interproximal sites of AL ≥ 4 mm or PD ≥ 5 mm on different teeth. Severe periodontitis was indicated by at least two interproximal sites with AL ≥ 6 mm on different teeth and one site with PD ≥ 5 mm. The absence of mild, moderate, or severe periodontitis signs was considered as no periodontitis.

### 2.4 Covariates

Multiple covariates that may influence the association of CircS with periodontitis were included, including age, sex, race, education, income-poverty ratio (PIR), marital status, smoking, alcohol consumption, physical activity (PA), and Healthy Eating Index-2015 (HEI-2015). Smoking status was categorized into never smokers (less than 100 lifetime cigarettes), former smokers (at least 100 lifetime cigarettes but quit at the time of the interview), and current smokers (current active smoking activity) based on participant responses on the smoking questionnaire (30). The Alcohol Use Questionnaire provided participants’ drinking history and patterns, and we categorized participants into never drinkers (less than 12 drinks in their lifetime), former drinkers (at least 12 drinks in their lifetime but abstained from alcohol in the last year), and current drinkers (further categorized as mild, moderate, and heavy drinkers based on sex-specific daily alcohol consumption) based on previous research (31). PA was categorized as no, moderate, and vigorous physical activity based on participants’ self-reports on the questionnaire (32). The HEI-2015 is an important reflection of dietary quality with nine adequate intake and four moderate intake food fractions, as assessed by dietary recall data and the food patterns equivalents data (33).

### 2.5 Selection of mediating variables

Previous studies have suggested that lipid profiles, oxidative stress and systemic inflammation may be involved in the pathogenesis of periodontitis (34–36). We included several variables that potentially mediate the association between CircS and periodontitis, including TG, total cholesterol (TC), HDL-C, low-density lipoprotein cholesterol (LDL-C), serum vitamin D, albumin (ALB), uric acid (UA), C-reactive protein (CRP), white blood cell (WBC) count, neutrophil count, and systemic immune-inflammation index (SII). SII = (platelet count × neutrophil count)/lymphocyte count. Blood cell counts were derived from parameters related to complete blood counts as measured by the Beckman Coulter DxH 800 method.

### 2.6 Statistical analysis

The statistical analyses incorporated appropriate sampling weights as per the NHANES analytic guidelines (37). This approach addressed the complex survey design and yielded national estimates. We first analyzed trends in the prevalence of CircS and periodontitis over the survey period. We carried out baseline analysis of participants by periodontitis status. Continuous variables are presented as mean ± standard error and assessed using weighted *t* test. Categorical variables are given as count (percentage) and examined via weighted chi-square test. We performed a univariate regression analysis of the covariates affecting periodontitis. We constructed several multivariable logistic regression models for examining the associations of CircS and its components with the odds of periodontitis in the general population and calculated the odds ratio (OR) and 95% confidence interval (CI). The crude model did not adjust for any covariates. Model 1 partially adjusted for age, sex, and race. Model 2 was additionally adjusted for education, PIR, marital status, smoking, alcohol consumption, PA, and HEI-2015 based on model 1. Fully adjusted RCS was applied to elucidate patterns of nonlinear or linear dose–response association between number of CircS components and prevalence of periodontitis. We chose appropriate knots for smooth curve fitting of the RCS model and computed the p for nonlinear values. In mediation analyses, we explored whether CircS indirectly mediated the association with periodontitis through selected mediator variables. The total effect of CircS on periodontitis consisted of the direct effect (DE) of CircS and the indirect effect (IE) of the mediating variables. If the mediating effect of a variable was statistically significant, we calculated the proportion mediated by the individual’s mediating variable in the total effect. Stratified analyses were employed to explore the heterogeneity of the association between CircS and periodontitis across subgroups and to identify moderators influencing this association through interaction tests. All analyses were executed in R version 4.2.3, with statistical significance set at *p* < 0.05.

## 3 Results

### 3.1 Baseline characteristics

**Figure 2:**
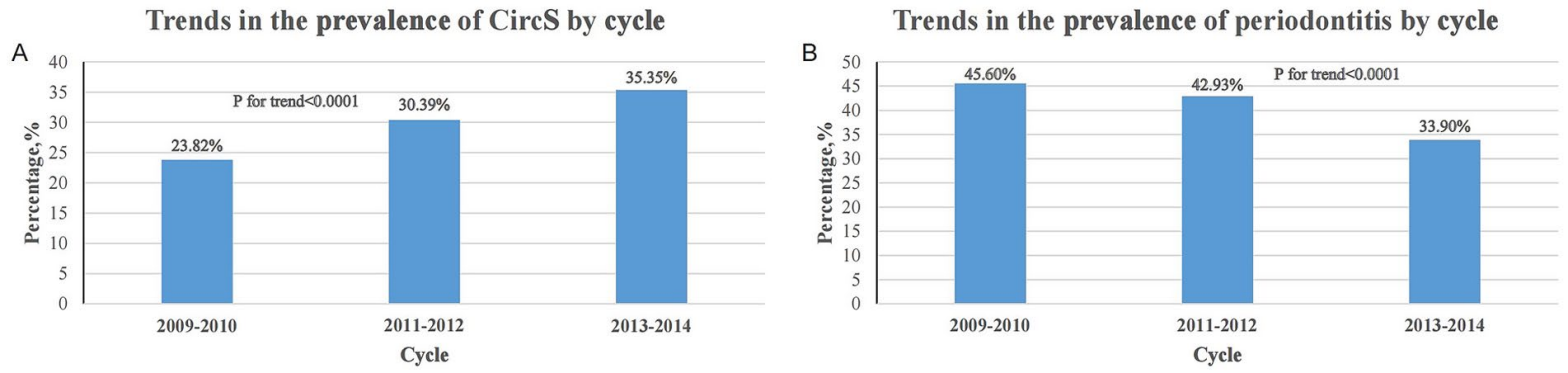
Trends in the prevalence of CircS and periodontitis over the survey period. **(A)** Trends in the prevalence of CircS across cycles from 2009 to 2014. **(B)** Trends in the prevalence of periodontitis across cycles from 2009 to 2014.

We found an increasing trend in the prevalence of CircS over time (23.82% in 2009–2010 to 35.35% in 2013–2014) (p for trend < 0.0001), whereas there was a significant decreasing trend in the prevalence of periodontitis (45.60% in 2009–2010 to 33.90% in 2013–2014) (p for trend <0.0001) (Figure 2). A total of 3,738 participants had periodontitis. Compared to patients without periodontitis, participants with periodontitis were older, had lower PIR and HEI-2015, and were more likely to be male, not of non-Hispanic White ethnicity, single, ≤high school educated, former/current smokers, former/heavy drinkers, and vigorously PA-involved. In addition, periodontitis participants had a higher prevalence of CircS and its components including elevated TG, reduced HDL-C, hyperglycemia, hypertension, and short sleep duration (Table 1).

**Table 1:** Baseline analysis according to periodontitis status.

Variable	Total (*n* = 7,555)	No-Periodontitis (*n* = 3,817)	Periodontitis (*n* = 3,738)	*p* value
Age	51.090 ± 0.264	48.419 ± 0.328	54.953 ± 0.361	<0.0001
PIR	3.249 ± 0.053	3.572 ± 0.056	2.782 ± 0.060	<0.0001
HEI-2015	55.875 ± 0.243	56.796 ± 0.350	54.541 ± 0.258	<0.0001
Sex				<0.0001
Male	3,737 (49.093)	1,549 (42.664)	2,188 (58.393)	
Female	3,818 (50.907)	2,268 (57.336)	1,550 (41.607)	
Race				<0.0001
Mexican American	1,011 (7.154)	372 (4.947)	639 (10.346)	
Non-Hispanic Black	1,521 (9.825)	620 (7.519)	901 (13.160)	
Non-Hispanic White	3,544 (72.102)	2030 (77.250)	1,514 (64.654)	
Other Hispanic	694 (4.727)	349 (4.277)	345 (5.378)	
Other Race	785 (6.192)	446 (6.006)	339 (6.461)	
Marital Status				<0.0001
Non-single	4,978 (70.669)	2,630 (74.534)	2,348 (65.079)	
Single	2,577 (29.331)	1,187 (25.466)	1,390 (34.921)	
Education				<0.0001
<High school	583 (3.967)	168 (2.109)	415 (6.656)	
High school	2,572 (29.813)	980 (22.423)	1,592 (40.504)	
>High school	4,400 (66.220)	2,669 (75.468)	1731 (52.841)	
Smoking				<0.0001
Never	4,219 (56.480)	2,484 (64.968)	1735 (44.201)	
Former	1985 (27.217)	890 (24.809)	1,095 (30.701)	
Now	1,351 (16.303)	443 (10.223)	908 (25.099)	
Drinking				<0.0001
Never	944 (9.618)	466 (9.579)	478 (9.675)	
Former	1,326 (14.639)	538 (11.879)	788 (18.631)	
Mild	2,792 (40.283)	1,521 (42.985)	1,271 (36.374)	
Moderate	1,181 (18.201)	704 (20.080)	477 (15.483)	
Heavy	1,312 (17.259)	588 (15.477)	724 (19.838)	
PA				<0.0001
No	4,521 (57.449)	2,340 (59.477)	2,181 (54.515)	
Moderate	1,612 (22.606)	822 (22.667)	790 (22.519)	
Vigorous	1,422 (19.945)	655 (17.856)	767 (22.966)	
Obesity				0.091
No	3,350 (43.560)	1707 (44.803)	1,643 (41.762)	
Yes	4,205 (56.440)	2,110 (55.197)	2095 (58.238)	
Elevated TG				<0.001
No	4,203 (55.479)	2,276 (58.891)	1927 (50.543)	
Yes	3,352 (44.521)	1,541 (41.109)	1811 (49.457)	
Reduced HDL-C				<0.0001
No	4,391 (59.519)	2,348 (62.299)	2043 (55.496)	
Yes	3,164 (40.481)	1,469 (37.701)	1,695 (44.504)	
Hyperglycemia				<0.0001
No	4,934 (67.638)	2,683 (71.713)	2,251 (61.742)	
Yes	2,621 (32.362)	1,134 (28.287)	1,487 (38.258)	
Hypertension				<0.0001
No	3,993 (56.973)	2,332 (63.403)	1,661 (47.671)	
Yes	3,562 (43.027)	1,485 (36.597)	2077 (52.329)	
Depression				0.06
No	6,931 (92.955)	3,516 (93.637)	3,415 (91.968)	
Yes	624 (7.045)	301 (6.363)	323 (8.032)	
Short sleep duration				<0.001
No	4,632 (65.282)	2,405 (67.627)	2,227 (61.891)	
Yes	2,923 (34.718)	1,412 (32.373)	1,511 (38.109)	
CircS				<0.0001
No	5,161 (70.034)	2,769 (73.833)	2,392 (64.538)	
Yes	2,394 (29.966)	1,048 (26.167)	1,346 (35.462)	

Continuous variables are presented as mean ± standard error and assessed using weighted *t* test. Categorical variables are given as count (percentage) and examined via weighted chi-square test.

### 3.2 Univariate analysis of covariates affecting periodontitis

Univariate regression analyses indicated that age, female, non-Hispanic White/other Hispanic/other race (Mexican American as ref), single, PIR, high school/>high school education (<high school as ref), former/current smokers (never-smokers as ref), former drinking (never-drinking as ref), vigorous PA (no PA as ref), and HEI-2015 were all associated with periodontitis (Supplementary Table S1).

### 3.3 Association of CircS with periodontitis

**Figure 3:**
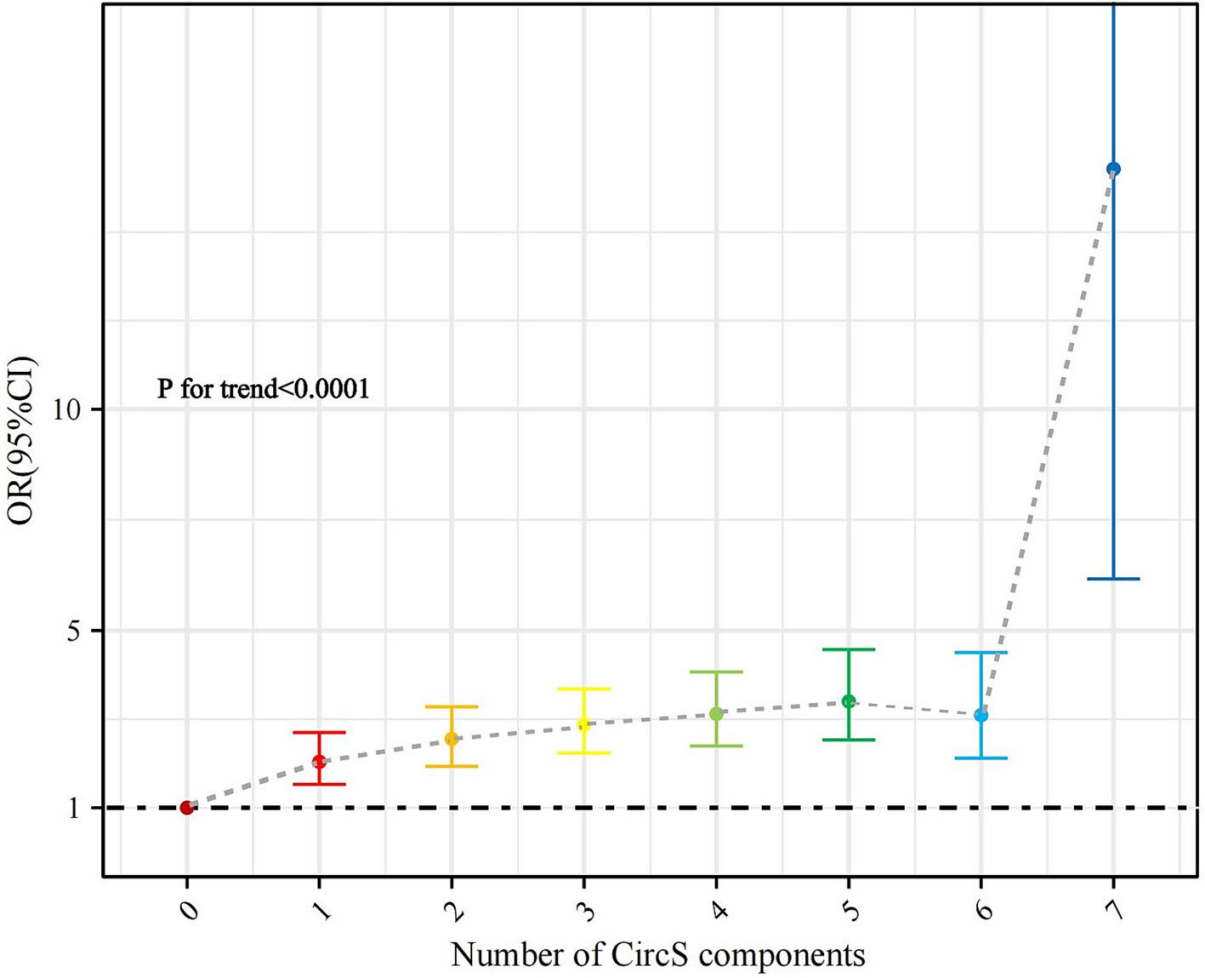
Association of the number of CircS components with periodontitis (with participants without any component as reference).

There was a trend toward a significant increase in the odds of periodontitis as the number of CircS components met by the participants increased (p for trend < 0.0001) (Figure 3). The prevalence of periodontitis was significantly higher (OR 14.594, *p* < 0.0001) in participants who fulfilled all 7 components compared to those who did not have the CircS component (Supplementary Table S2).

CircS was significantly associated with the prevalence of periodontitis in both the crude model and Model 1 (*p* < 0.0001). In the fully adjusted model 2, we found that CircS remained significantly associated with periodontitis (OR 1.509, 95% CI 1.326–1.716, *p* < 0.0001). A greater number of components did not significantly increase the prevalence of periodontitis after meeting the CircS diagnosis (fulfilling five components: OR = 1.091, *p* = 0.394; fulfilling 6–7 components: OR = 1.159, *p* = 0.315) (Table 2). Analysis of specific components showed central obesity (OR 1.232, *p* = 0.011), elevated TG (OR 1.305, *p* = 0.004), reduced HDL-C (OR 1.289, *p* < 0.0001), hyperglycemia (OR 1.494, *p* < 0.0001), hypertension (OR 1.872, *p* < 0.0001), depression (OR 1.374, *p* = 0.029), and short sleep duration (OR 1.224, *p* = 0.002) were all significantly associated with periodontitis (Table 3).

**Table 2:** Association of CircS with prevalence of periodontitis.

	Crude Model OR (95%CI)	*p*-value	Model 1 OR (95%CI)	*p*-value	Model 2 OR (95%CI)	*p*-value
CircS						
No	Ref	Ref	Ref	Ref	Ref	Ref
Yes	1.550 (1.363, 1.763)	<0.0001	1.530 (1.341, 1.746)	<0.0001	1.509 (1.326, 1.716)	<0.0001
Number of CircS components						
4	Ref	Ref	Ref	Ref	Ref	Ref
5	1.079 (0.891, 1.307)	0.427	1.087 (0.886, 1.335)	0.414	1.091 (0.889, 1.338)	0.394
6–7	1.059 (0.798, 1.407)	0.684	1.151 (0.857, 1.546)	0.342	1.159 (0.865, 1.552)	0.315

The crude model did not adjust for any covariates. Model 1 partially adjusted for age, sex, and race. Model 2 was additionally adjusted for education, PIR, marital status, smoking, alcohol consumption, PA, and HEI-2015 based on model 1.

**Table 3:** Association of CircS components with periodontitis.

	Crude Model OR (95%CI)	*p*-value	Model 1 OR (95%CI)	*p*-value	Model 2 OR (95%CI)	*p*-value
Central obesity						
No	Ref	Ref	Ref	Ref	Ref	Ref
Yes	1.132 (0.980, 1.308)	0.091	1.264 (1.075, 1.486)	0.006	1.232 (1.051, 1.444)	0.011
Elevated TG						
No	Ref	Ref	Ref	Ref	Ref	Ref
Yes	1.402 (1.174, 1.674)	<0.001	1.314 (1.099, 1.572)	0.004	1.305 (1.091, 1.561)	0.004
Reduced HDL-C						
No	Ref	Ref	Ref	Ref	Ref	Ref
Yes	1.325 (1.191, 1.474)	<0.0001	1.312 (1.175, 1.466)	<0.0001	1.289 (1.158, 1.435)	<0.0001
Hyperglycemia						
No	Ref	Ref	Ref	Ref	Ref	Ref
Yes	1.571 (1.422, 1.736)	<0.0001	1.488 (1.347, 1.644)	<0.0001	1.494 (1.355, 1.648)	<0.0001
Hypertension						
No	Ref	Ref	Ref	Ref	Ref	Ref
Yes	1.902 (1.639, 2.206)	<0.0001	1.862 (1.606, 2.160)	<0.0001	1.872 (1.615, 2.170)	<0.0001
Depression						
No	Ref	Ref	Ref	Ref	Ref	Ref
Yes	1.285 (0.989, 1.670)	0.06	1.434 (1.074, 1.914)	0.016	1.374 (1.035, 1.824)	0.029
Short sleep duration						
No	Ref	Ref	Ref	Ref	Ref	Ref
Yes	1.286 (1.136, 1.457)	<0.001	1.252 (1.110, 1.413)	<0.001	1.224 (1.084, 1.380)	0.002

The crude model did not adjust for any covariates. Model 1 partially adjusted for age, sex, and race. Model 2 was additionally adjusted for education, PIR, marital status, smoking, alcohol consumption, PA, and HEI-2015 based on model 1.

### 3.4 RCS analysis of the association between the number of CircS components and periodontitis

**Figure 4:**
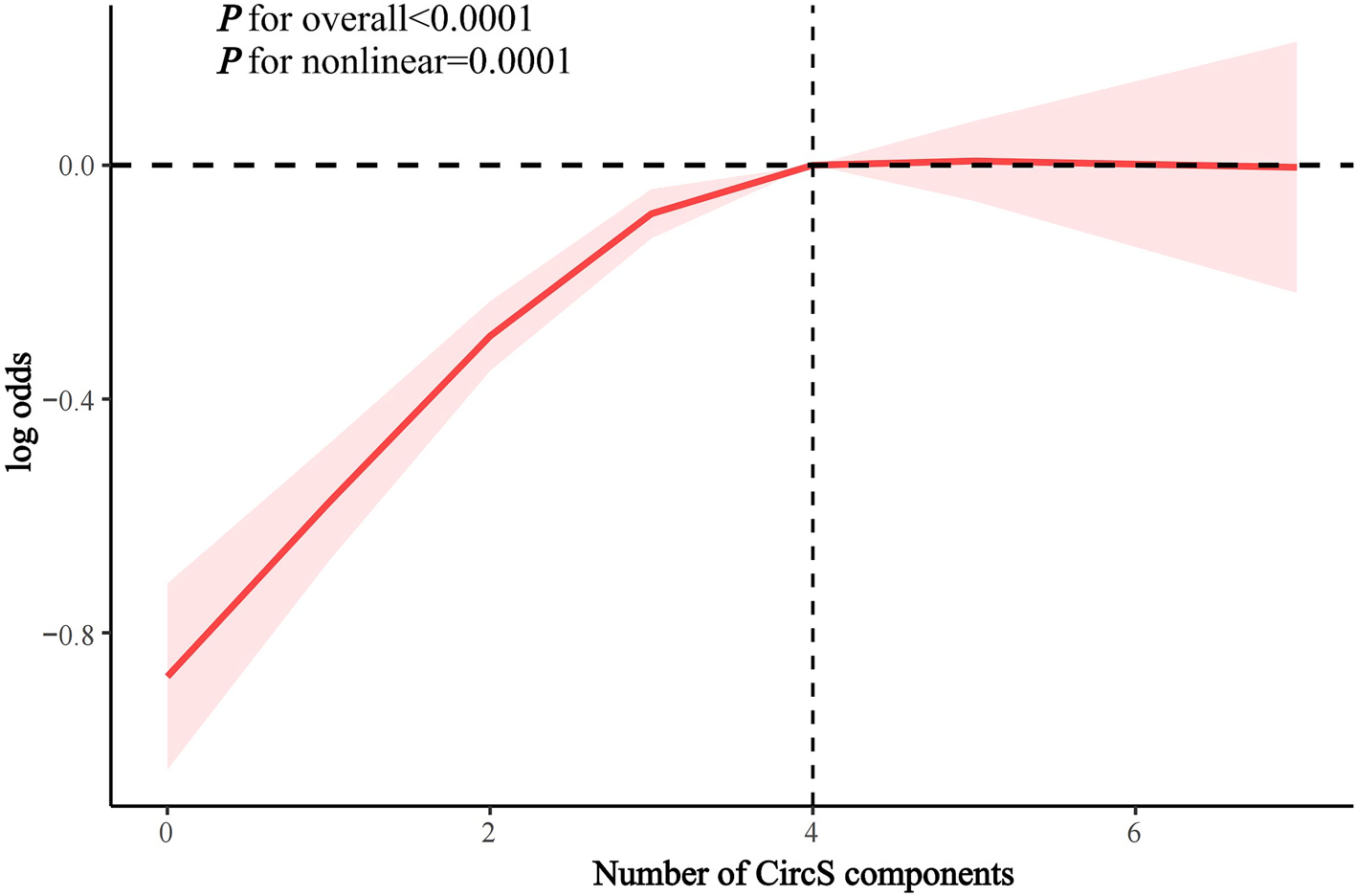
RCS analysis of the association between the number of CircS components and periodontitis.

RCS analysis showed a nonlinear association between the number of CircS components and the odds of periodontitis (inflection point = 4, p for nonlinearity = 0.0001) (Figure 4). There was a significant association between CircS and periodontitis when the number of components was <4 (OR 1.323, 95% CI 1.240–1.412, p < 0.0001), while there was no significant association when the number of components was ≥4 (Table 4).

**Table 4:** Threshold effect analysis of the association between the number of CircS components and periodontitis.

Character	OR 95% CI	*p*	p for interaction
Number of CircS components			0.011
<4	1.323 (1.240, 1.412)	**<0.0001**	
≥4	1.106 (0.978, 1.251)	0.106	

The bolded value (<0.0001) indicates statistical significance.

### 3.5 Mediation analysis

**Figure 5:**
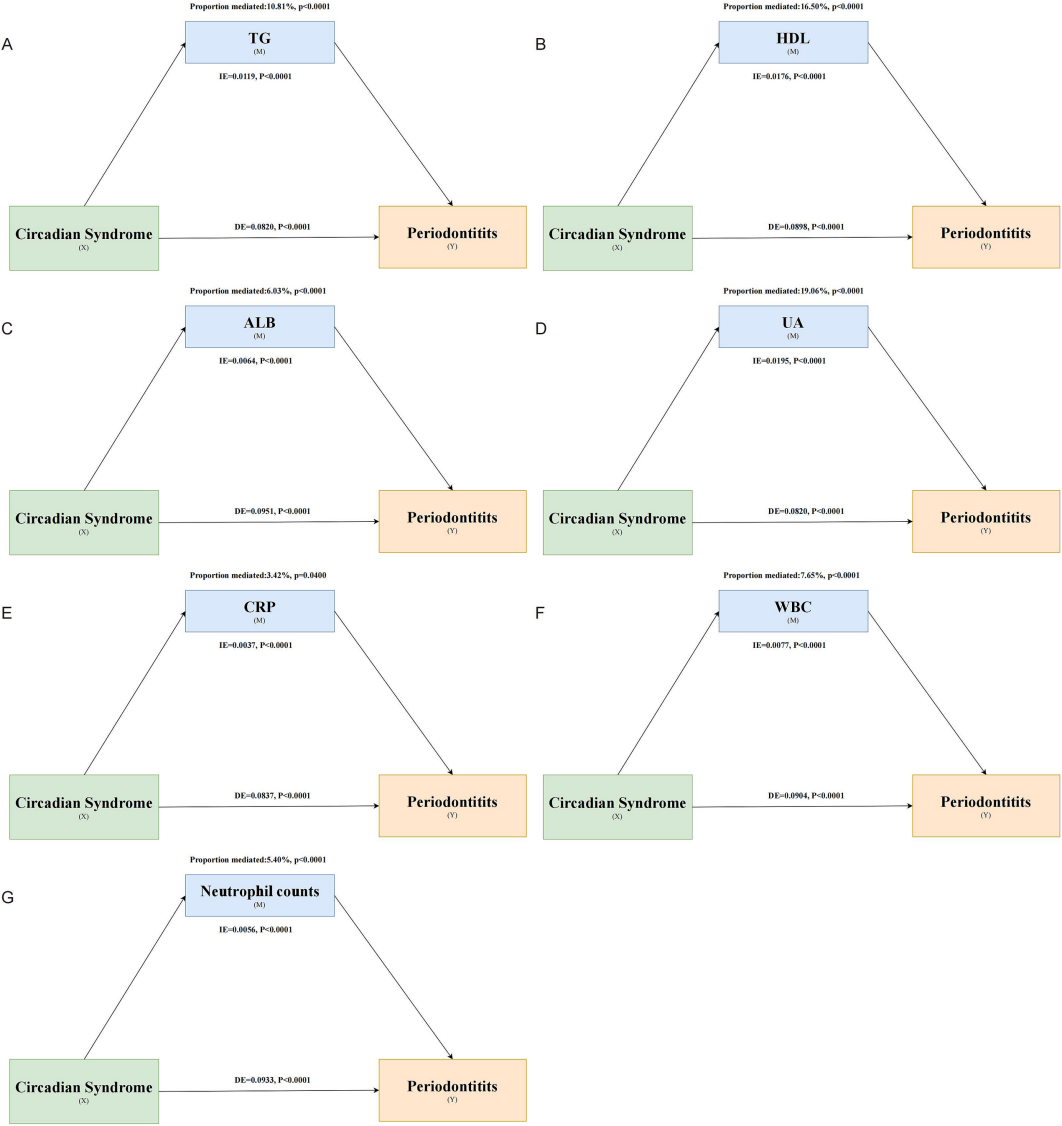
Mediating role of various biomarkers in the association between CircS and periodontitis. Panels **(A–G)** represent the mediating effects of TG, HDL-C, ALB, UA, CRP, WBC, and neutrophil count, respectively.

Mediation analysis showed that TG (proportion mediated 10.81%), HDL-C (proportion mediated 16.50%), ALB (proportion mediated 6.03%), UA (proportion mediated 19.06%), CRP (proportion mediated 3.42%), WBC (proportion mediated 7.65%), and neutrophil counts (proportion mediated 5.40%) significantly mediated the association between CircS and periodontitis (Figure 5). TC, LDL-C, vitamin D, and SII did not significantly mediate this association (Supplementary Tables S3–S6).

### 3.6 Stratified analysis

**Figure 6:**
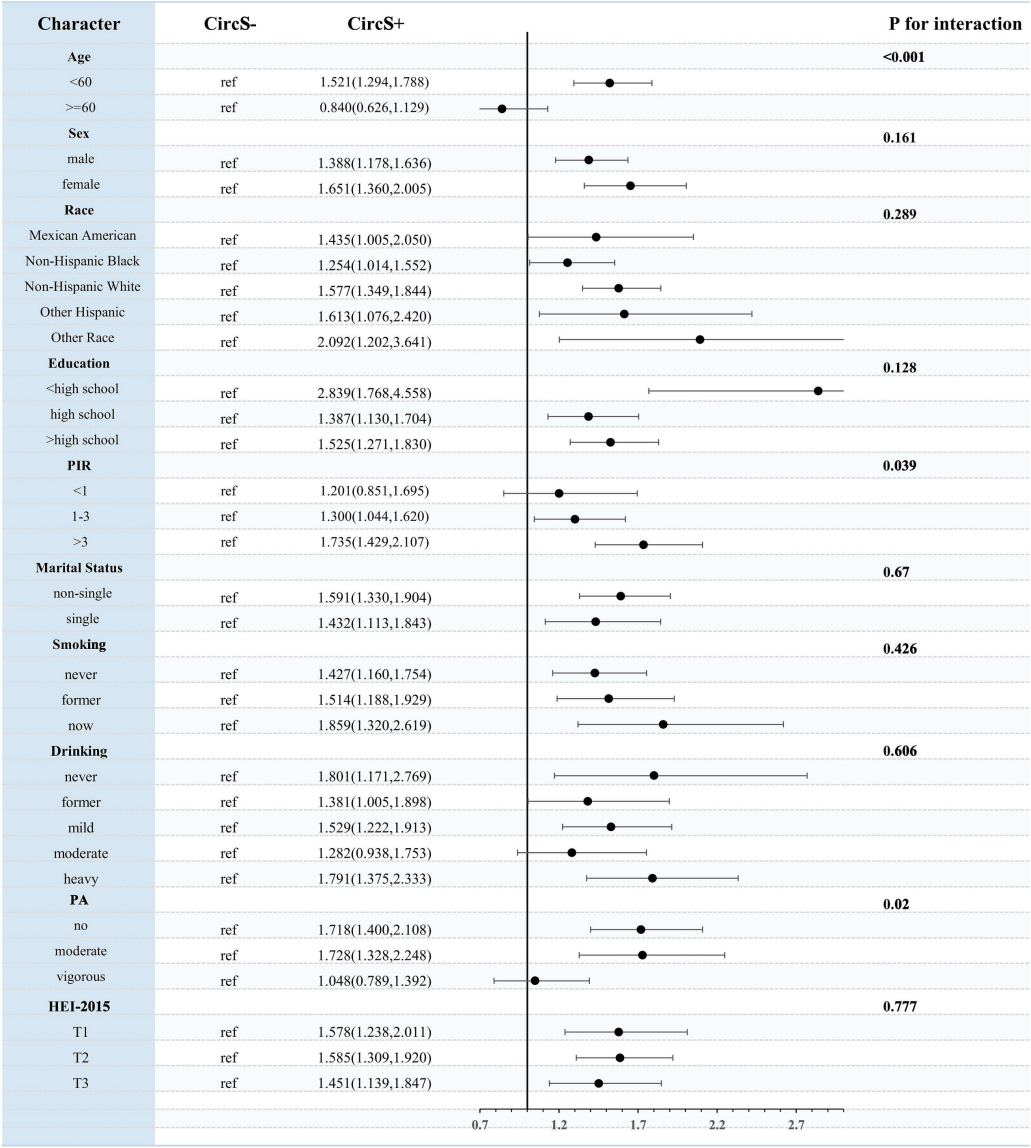
Stratified analysis of the association between CircS and periodontitis.

Stratified analyses indicated that age (p for interaction < 0.001), PIR (p for interaction = 0.039), and PA (p for interaction = 0.02) significantly influenced this association. This association was more significant in participants <60 years, with PIR > 3, and with non-vigorous PA (Figure 6).

## 4 Discussion

### 4.1 Main findings

In a national cross-sectional analysis, CircS and its components were all significantly associated with the prevalence of periodontitis, independently of all confounders. Compared to individuals without CircS, the presence of CircS was associated with a 50.9% increase in the prevalence of periodontitis. A higher number of components was associated with significantly increased odds of periodontitis compared with participants without CircS components. However, an increase in the number of components after meeting the diagnosis of CircS was not associated with an increased odd of periodontitis. RCS analysis showed a nonlinear association between the number of components and periodontitis, with a positive association existing when the number of components was <4. Mediation analysis showed that TG, HDL-C, ALB, UA, CRP, WBC, and neutrophil count significantly mediated the association of CircS with periodontitis. Age, PIR, and PA significantly interacted with this association.

### 4.2 Comparison with previous studies

To the best of our knowledge, this is the first time that the association of CircS with periodontitis in the general population has been explored through a large national survey. The circadian rhythm system is a major regulator of human metabolic health (38). Circadian rhythm disruption is associated with multiple components and outcomes of MetS, such as obesity, type 2 diabetes, and CVD, which can lead to insulin resistance, dysglycemia, and dyslipidemia, increasing the risk of MetS (39). A growing body of evidence suggests that circadian rhythm disruption is also closely associated with a variety of other chronic diseases, including sleep disorders and depression (21). Therefore, given that circadian rhythm disruption may be an important underlying etiology of MetS, the concept of CircS was proposed and added to MetS the assessment of sleep duration and depression (21, 22). Previous cross-sectional studies derived from NHANES have shown that CircS was significantly and positively associated with the odds of developing multiple chronic noncommunicable diseases, including CVD (23), gallbladder stones (28), chronic diarrhea (27), psoriasis (40), and frailty (41). Evidence from animal studies suggests that circadian rhythm disruption contributes to the onset and progression of periodontitis by modulating the expression of circadian key genes such as BMAL1, thereby affecting oxidative stress, inflammation, and apoptosis in periodontal tissues (17, 18, 42). Our study demonstrated for the first time that CircS was significantly associated with periodontitis in the general population, suggesting that circadian rhythm disruption has an important implication in periodontal health and emphasizing that CircS may serve as an emerging risk factor and management target for periodontitis. In addition, our findings revealed that the association between CircS and periodontitis was more pronounced in specific subgroups, including participants <60 years of age, PIR > 3, and participants with non-vigorous PA involvement, emphasizing the need for extra attention to this association in specific populations.

As a basis for CircS, a large body of literature has shown a bidirectional association between MetS and periodontitis. A meta-analysis pooling 14 studies showed that moderate (OR 1.26, 95%CI 2.10–5.37) and severe periodontitis (OR 1.50, 95%CI 1.28–1.71) were significantly associated with the odds of MetS (11). Sayeed et al. demonstrated a bidirectional association between MetS and periodontitis by meta-analysis (MetS-periodontitis: OR 1.566, 95% CI 1.359–1.806; periodontitis-MetS: OR 1.604, 95% CI 1.370–1.879), although this bi-directional association disappeared in the female subgroup (12). In a meta-analysis including 38 studies, Campos et al. showed that most of the MetS components were significantly associated with periodontitis (hyperglycemia: OR 1.18; hypertension: OR 1.11; low HDL-C: OR 1.16; obesity: OR 1.08) (13). In addition, there was a dose–response association between the number of MetS components and the odds of periodontitis (ORs of 1.14, 1.52, 1.79, and 2.02 for components ranging from 1, 2, 3, to 4–5) (13). Earlier meta-analyses similarly showed that MetS was significantly associated with periodontitis (43–45).

Several observational studies have shown an association between short sleep duration and the development of periodontitis; however inconsistent findings have been noted. A recent meta-analysis incorporating 11 cross-sectional analyses demonstrated that short sleep duration was not associated with the occurrence of periodontitis or severe periodontitis (periodontitis: OR 1.04, 95% CI 0.83–1.29; severe periodontitis: OR 0.94, 95% CI 0.75–1.16) (46). However, when short sleep duration was defined as ≤5 h, a significant association between sleep deprivation and periodontitis was found (OR 1.41, 95% CI 1.33–1.51) (46). A cross-sectional study utilizing NHANES 2005–2020 suggested that insufficient sleep (<7 h) was significantly associated with moderate/severe periodontitis (OR 1.15, 95% CI 1.01–1.30, *p* = 0.0298) (47). Depression may influence the development of periodontitis through neurobiological, neurobehavioral, and immune-microbiome interactions (48). Some observational studies have also suggested a possible association between depression and periodontitis, however there is also significant controversy. A meta-analysis of 7 cross-sectional analyses showed no significant association between depression and periodontitis (OR 1.03, 95% CI 0.75–1.41) (49). Another meta-analysis of three cross-sectional studies similarly showed no significant association between depression and periodontal disease (OR 0.78, 95% CI 0.44–1.99) (50). However, another meta-analysis showed that depression was significantly associated with the development of chronic periodontitis (OR 1.61, 95% CI 1.16–2.23) (51). A birth cohort study showed the presence of depressive symptoms was associated with an increased risk of periodontitis (relative risk 1.19) (52). A cross-sectional analysis from South Korea showed diagnosed depression was associated with odds of periodontitis (OR 1.772, 95% CI 1.328–2.364), while self-reported depression was not significantly associated (53). These studies suggest controversial findings on the association between depression and the development of periodontitis, and we speculate that this association may be influenced by the means of depression assessment and the diagnosis of periodontitis. Our findings indicated that depression as a component of CircS was significantly associated with periodontitis. Cohort studies are needed to validate the plausibility of this association, given the cross-sectional nature of the design of most studies.

### 4.3 Potential mechanisms

Evidence from animal studies suggests that circadian rhythm disruption contributes to the onset and progression of periodontitis by modulating the expression of circadian key genes such as BMAL1, thereby affecting oxidative stress, inflammation, and apoptosis in periodontal tissues (17, 18, 42). Inadequate sleep duration and depressive symptoms as unique components of CircS are recognized as important consequences of circadian rhythm disruption. Inadequate sleep duration may significantly affect the body’s innate immunity and increase systemic inflammation levels, with important implications for periodontal health and disease (54).

Systemic inflammation is an important disease hallmark of MetS, and activation of circulating immune cells and release of proinflammatory cytokines can exacerbate the inflammatory response in periodontal tissues and promote the development of periodontitis (55, 56). Patients with MetS often suffer from a state of oxidative stress, which impairs the ability of periodontal tissues to respond to bacterial attack and leads to an increased risk of periodontitis (55). Elevated reactive oxygen species are associated with chronic activation of inflammatory mediators in the gingiva, leading to alveolar bone destruction and exacerbation of periodontitis features (57). MetS may also affect the oral microenvironment and alter the composition of the oral microbiota, increasing susceptibility to periodontitis (57). In addition, disturbances in the serum lipid profile may also have an important role in the pathogenesis of periodontitis (34), although there is a lack of relevant studies linking dyslipidemia to the association of MetS with periodontitis. Overall, lipid profiles, inflammation, oxidative stress, altered host responses, and the oral microbiome have potential roles in the association of MetS and periodontitis (58). The core assumption of the mediation analysis is the temporal sequence of “CircS → mediating variable → periodontitis,” but cross-sectional data cannot directly verify this temporal sequence. The current mediation analysis results only suggest potential mediating pathways, which need to be further verified through longitudinal studies to establish causal chains. Given that most of the current studies examining the association between selected variables (such as blood lipids and CRP) and periodontitis are observational non-cohort studies, these mediating effects require support from longitudinal studies and mechanism studies.

### 4.4 Study strengths and limitations

Some potential advantages exist in our study. It is a nationally representative population-based study with a large sample, making the findings potentially generalizable. We adequately considered confounding factors to reduce study bias. We provide preliminary insights into the mechanisms underlying this association through mediation analyses suggesting that CircS may mediate the association with periodontitis by modulating serum lipid profiles, oxidative stress, and systemic inflammation. These findings have important clinical implications, suggesting that CircS may be an independent risk factor for periodontitis and may reduce the occurrence of periodontitis by altering circadian rhythm disruption in individuals. However, our study has some noteworthy limitations. This was a cross-sectional study and therefore could not assess the temporality and causality of associations and was subject to residual confounding. Sleep duration was assessed by self-report and may be influenced by recall bias. In addition, these findings were based on NHANES and whether they can be generalized to other settings or national populations requires future validation.

## 5 Conclusion

The presence of CircS and its components was significantly associated with periodontitis in the general population. A higher number of components was associated with increased odds of periodontitis compared to the absence of any CircS component. However, the number of CircS components was nonlinearly associated with periodontitis. Positive association existed when the number of components was <4, whereas an increase in the number of components after fulfilling the diagnosis of CircS was not associated with the odds of periodontitis. Several serum lipids, oxidative stress, and systemic inflammatory markers mediated the association of CircS with periodontitis. This association was more pronounced in participants <60 years of age, PIR > 3, and non-vigorous PA. These findings suggest that CircS may be an independent risk factor for periodontitis and mediate this association through serum lipids, systemic inflammation, and oxidative stress. This study suggests that CircS screening could be incorporated into periodontal disease risk assessment systems, particularly for individuals under 60 years of age, those with high incomes, and those who lack physical activity. However, cross-sectional designs cannot rule out reverse causality and require validation through longitudinal studies.

## Data Availability

Publicly available datasets were analyzed in this study. This data can be found here: https://www.cdc.gov/nchs/nhanes/.
