# Supplementary material for: Serum lipids, oxidative stress, and systemic inflammation mediate the association between circadian syndrome and periodontitis
Source: Front Nutr. 2025 Jul 2;12:1622348. doi: 10.3389/fnut.2025.1622348 (PMC12263390; doi:10.3389/fnut.2025.1622348)
Supplement: Supplementary file 1 [file Table_1.docx]

**Table S1**. Univariate analysis of covariates affecting periodontitis.

| Character | OR 95% CI | P |
| --- | --- | --- |
| Age | 1.039(1.033,1.045) | <0.0001 |
| Sex |  |  |
| male | ref | ref |
| female | 0.530(0.472,0.595) | <0.0001 |
| Race |  |  |
| Mexican American | ref | ref |
| Non-Hispanic Black | 0.837(0.664,1.055) | 0.128 |
| Non-Hispanic White | 0.400(0.319,0.503) | <0.0001 |
| Other Hispanic | 0.601(0.477,0.758) | <0.0001 |
| Other Race | 0.514(0.391,0.676) | <0.0001 |
| Marital Status |  |  |
| non-single | ref | ref |
| single | 1.570(1.383,1.784) | <0.0001 |
| PIR | 0.730(0.698,0.764) | <0.0001 |
| Education |  |  |
| <high school | ref | ref |
| high school | 0.572(0.459,0.714) | <0.0001 |
| >high school | 0.222(0.173,0.285) | <0.0001 |
| Smoke |  |  |
| never | ref | ref |
| former | 1.819(1.568,2.110) | <0.0001 |
| now | 3.608(3.074,4.235) | <0.0001 |
| Drinking |  |  |
| never | ref | ref |
| former | 1.553(1.198,2.013) | 0.001 |
| mild | 0.838(0.629,1.115) | 0.219 |
| moderate | 0.763(0.569,1.024) | 0.07 |
| heavy | 1.269(0.921,1.748) | 0.141 |
| Physical activity |  |  |
| no | ref | ref |
| moderate | 1.084(0.956,1.229) | 0.203 |
| vigorous | 1.403(1.203,1.636) | <0.0001 |
| HEI | 0.987(0.983,0.992) | <0.0001 |

**Table S2**. Association between different number of CircS components (with number 0 as reference) and prevalence of periodontitis.

| Character | P | OR(95% CI) |
| --- | --- | --- |
| Number of CircS components |  |  |
| 0 | ref | ref |
| 1 | <0.0001 | 1.990(1.505, 2.632) |
| 2 | <0.0001 | 2.390(1.844, 3.098) |
| 3 | <0.0001 | 2.761(2.164, 3.523) |
| 4 | <0.0001 | 3.103(2.395, 4.021) |
| 5 | <0.0001 | 3.417(2.550, 4.577) |
| 6 | <0.0001 | 3.022(2.066, 4.421) |
| 7 | <0.0001 | 14.594(5.447,39.097) |

**Table S3**. Mediating effects of TC in the association of CircS with periodontitis.

| **Effect** | **Estimate** | **Lower** | **Upper** | **P** | **Proportion** |
| --- | --- | --- | --- | --- | --- |
| Indirect | 0.0013 | -0.0008 | 0.0033 | 0.4000 | 1.4001 |
| Direct | 0.1003 | 0.0722 | 0.1246 | <.0001 | 98.5999 |
| Total | 0.1016 | 0.0749 | 0.1258 | <.0001 | 100.0000 |

**Table S4**. Mediating effects of LDL-C in the association of CircS with periodontitis.

| **Effect** | **Estimate** | **Lower** | **Upper** | **P** | **Proportion** |
| --- | --- | --- | --- | --- | --- |
| Indirect | -0.0005 | -0.0046 | 0.0023 | 0.8800 | -0.3763 |
| Direct | 0.0899 | 0.0490 | 0.1252 | <.0001 | 100.3763 |
| Total | 0.0894 | 0.0505 | 0.1243 | <.0001 | 100.0000 |

**Table S5**. Mediating effects of vitamin D in the association of CircS with periodontitis.

| **Effect** | **Estimate** | **Lower** | **Upper** | **P** | **Proportion** |
| --- | --- | --- | --- | --- | --- |
| Indirect | -0.0018 | -0.0041 | -0.0001 | 0.0800 | -1.6956 |
| Direct | 0.1024 | 0.0746 | 0.1264 | <.0001 | 101.6956 |
| Total | 0.1006 | 0.0732 | 0.1247 | <.0001 | 100.0000 |

**Table S6**. Mediating effects of SII in the association of CircS with periodontitis.

| **Effect** | **Estimate** | **Lower** | **Upper** | **P** | **Proportion** |
| --- | --- | --- | --- | --- | --- |
| Indirect | 0.0006 | -0.0002 | 0.0016 | 0.1200 | 0.5244 |
| Direct | 0.0987 | 0.0710 | 0.1227 | <.0001 | 99.4756 |
| Total | 0.0993 | 0.0711 | 0.1236 | <.0001 | 100.0000 |
